# lncRNAs-EZH2 interaction as promising therapeutic target in cutaneous melanoma

**DOI:** 10.3389/fmolb.2023.1170026

**Published:** 2023-05-31

**Authors:** Michal Wozniak, Malgorzata Czyz

**Affiliations:** Department of Molecular Biology of Cancer, Medical University of Lodz, Lodz, Poland

**Keywords:** epigenetics, EZH2, immunotherapy, lncRNAs, melanoma, PRC2, targeted therapy

## Abstract

Melanoma is the most lethal skin cancer with increasing incidence worldwide. Despite a great improvement of diagnostics and treatment of melanoma patients, this disease is still a serious clinical problem. Therefore, novel druggable targets are in focus of research. EZH2 is a component of the PRC2 protein complex that mediates epigenetic silencing of target genes. Several mutations activating EZH2 have been identified in melanoma, which contributes to aberrant gene silencing during tumor progression. Emerging evidence indicates that long non-coding RNAs (lncRNAs) are molecular “address codes” for EZH2 silencing specificity, and targeting lncRNAs-EZH2 interaction may slow down the progression of many solid cancers, including melanoma. This review summarizes current knowledge regarding the involvement of lncRNAs in EZH2-mediated gene silencing in melanoma. The possibility of blocking lncRNAs-EZH2 interaction in melanoma as a novel therapeutic option and plausible controversies and drawbacks of this approach are also briefly discussed.

## 1 Introduction

Cutaneous melanoma remains the most lethal of skin cancers and its incidence continues to increase worldwide (Sung et al., 2021; Siegel et al., 2022). Early stage melanomas can be successfully treated with surgery alone, whereas for late stages of disease diverse targeted therapies or immunotherapies are used. Despite the development of targeted drugs against BRAF^V600^ kinase and its target kinase MEK1/2 (Flaherty et al., 2012; Robert et al., 2015; Ascierto et al., 2016; Dummer et al., 2018), the majority of patients require additional therapy because of intrinsic or acquired resistance (Atkins et al., 2021). The immunotherapy with antibodies against immune checkpoint proteins cytotoxic T cell antigen 4 (CTLA-4) and programmed death receptor 1/ligand (PD-1/PD-L1) has greatly extended the lives of many melanoma patients, however, about 70% of patients show disease progression within 5 years (Atkins et al., 2021). Moreover, a subset of patients with autoimmune disorders, or organ transplant recipients are ineligible for melanoma immunotherapy. Therefore, novel druggable targets are still being sought to combat melanoma.

While the development of targeted therapies is mainly focused on genetic alterations, growing evidence indicates that aberrant epigenetic control of cell fate substantially contributes to carcinogenesis. Therefore, epigenetic mechanisms such as non-coding RNAs (nc-RNAs), DNA methylation and histone modifications have attracted attention of cancer research for last years (Sarkar et al., 2015; Moran et al., 2018; Santourlidis et al., 2022).

In this review we summarize the current knowledge on enhancer of zeste homolog 2 (EZH2), an epigenetic modifier with methyltransferase activity, and lncRNAs as modulators of EZH2 activity. We outline the involvement of lncRNAs-EZH2 interaction in cancer focusing on melanoma. Finally, targeting the lncRNAs-EZH2 interaction and plausible controversies regarding this approach as potential treatment modality are also briefly discussed.

## 2 lncRNAs in health and disease

Over 90% of mammalian genome is transcribed from DNA, but only up to 2% of transcripts code for proteins (ENCODE Project Consortium, 2012). Approximately 62%–75% of all transcripts is classified as non-coding RNAs (Djebali et al., 2012; Iyer et al., 2015). According to the most recent definition and classification (Mattick et al., 2023) lncRNAs are RNA molecules that are over 500 nucleotides long, bearing limited or no protein-coding abilities. However, they exhibit many of the characteristics of mRNAs: their genes are transcribed mostly by RNA polymerase II, most of lncRNA transcripts are 5′-capped, 3′-polyadenylated, and undergo splicing (Kapranov et al., 2007; Dinger et al., 2008; Guttman et al., 2009; Derrien et al., 2012).

The first non-coding RNA that resembled mRNAs in length and splicing structure but did not code for any protein was H19. It has been identified as a long non-coding RNA induced during the development of liver in mice (Pachnis et al., 1988; Brannan et al., 1990). Impressive progress in sequencing technologies coupled with new computational methods for assembling the transcriptome and predicting transcription sites have permitted to identify non-coding transcripts across many different cell types and tissues (Mortazavi et al., 2008; Cabili et al., 2011). Between 30 and 60 thousand of lncRNAs have been identified and annotated in human genome in recent years (Iyer et al., 2015; Hon et al., 2017) and this number is still growing. The huge amount of identified human lncRNAs, their extremely low expression levels and low sequence conservation when compared with mRNAs (Ponjavic et al., 2007; Cabili et al., 2011), have raised the concern that many of these transcripts may comprise a transcriptional noise with no functions, or are incidental by-products of transcription from enhancer regions (Struhl, 2007; De Santa et al., 2010). However, evidence from published scientific reports clearly indicates the *bona fide* role of lncRNA in health and disease (Nagano and Fraser, 2011; Spizzo et al., 2012; Policarpo et al., 2021; Mirzaei et al., 2022; Mitra et al., 2022). The expression of lncRNA is highly tissue- and disease-specific (Cabili et al., 2011; Lee et al., 2011), which suggests that lncRNAs play important role in the determination of cell fate and function of differentiated cells (Derrien et al., 2012; Yan et al., 2015). They retain evolutionary conservation with regards to their biological functions, despite rapid sequence evolution (Ulitsky et al., 2011; Hezroni et al., 2015). lncRNAs possess great potential to control and modulate crucial signaling pathways in human cells dependent on cellular localization: nuclear lncRNAs are involved in chromatin modifications, transcriptional regulation, and RNA processing, while cytoplasmic lncRNAs modulate mRNA stability or translation and various cellular signaling cascades (Batista and Chang, 2013). lncRNAs can also directly interact with proteins (Zhang et al., 2019), or act as sponges for miRNAs (López-Urrutia et al., 2019).

To date, many lncRNAs have been identified with a prominent role in melanoma progression (Sarkar et al., 2015; Wozniak and Czyz, 2021; Montico et al., 2022). Several lncRNAs, such as survival-associated mitochondrial melanoma specific oncogenic non-coding RNA (SAMMSON), MAPK inhibitor resistance associated transcript (MIRAT) or the lncRNAs SPRY4 intronic transcript 1 (SPRY4-IT1) reside mostly in cytoplasm, where they bind to various proteins and affect their stability and activity. SAMMSON has been positively associated with SOX10 transcription factor responsible for the development of neural and pigment cells that derive from the neural crest (Leucci et al., 2016). *SAMMSON* is colocalized with *MITF* and the amplification of both genes in approximately 10% of melanomas has been associated with poor prognosis (Garraway et al., 2005). Small fraction of SAMMSON resides in mitochondria, where it binds and stabilizes p32 protein, a master regulator of mitochondrial homeostasis and metabolism, triggering its oncogenic activity (Leucci et al., 2016). SPRY4-IT1 has been found upregulated in samples from melanoma patients, when compared to control melanocytes. Elevated level of SPRY4-IT1 significantly correlates with reduced overall survival of melanoma patients (Liu et al., 2016). SPRY4-IT1 regulates lipid metabolism by directly binding to lipin 2 enzyme that converts phosphatidates to diacylglycerols. Knockdown of SPRY4-IT1 has been shown to reduce proliferation, migration and invasiveness of melanoma cells (Khaitan et al., 2011) and alter a lipid metabolism, leading to cellular lipotoxicity and induction of apoptosis (Mazar et al., 2014). MIRAT has been demonstrated to bind and stabilize cytoplasmic scaffold protein IQ motif containing GTPase activating protein 1 (IQGAP1) that positively regulates the MAPK pathway in NRAS mutated melanomas (Sanlorenzo et al., 2018).

Cytosolic lncRNAs may also act as competing endogenous RNAs (ceRNAs), also called RNA sponges, as they bind miRNAs in accordance with their regions of sequence complementarity and block their activity, resulting in the promotion of translation of miRNA coding target transcripts (Salmena et al., 2011; Yoon et al., 2014). Therefore, lncRNAs may be considered as oncogenes, when their sponging activity induces the expression of oncogenes (e.g., BANCR), or tumor suppressors when they augment the expression of tumor suppressor genes (e.g., MEG3). BRAF-activated non-coding RNA (BANCR) is mostly melanoma and melanocyte-specific lncRNA upregulated in BRAF^V600^ melanomas compared to normal melanocytes. It has been shown that BANCR promotes melanoma by the activation of ERK1/2 and JNK kinases, which results in shorter overall survival of melanoma patients (Li et al., 2014). BANCR sponging activity towards miR-204-5p has been identified, and loss of functional miR-204-5p activates Notch2 oncogene (Cai et al., 2017). Maternally expressed gene 3 (MEG3) lncRNA, on the other hand, is a tumor suppressor downregulated in melanoma and its low expression has correlated with poor prognosis (Long and Pi, 2018). It has been found that re-expression of MEG3 limits EMT-like phenotype in melanoma through upregulation of E-cadherin by targeting miR-21 (Wu et al., 2020) and miR-499-5p, which negatively regulates tumor suppressor CYLD lysine 63 deubiquitinase (Long and Pi, 2018).

Recently published papers indicate the involvement of lncRNAs in tumor microenvironment maintenance and immunotherapy efficacy in melanoma (Zhou et al., 2022; Xiong et al., 2023). The upregulation of SMG7-AS1 has been implicated in melanoma progression, since this lncRNA controls apoptosis cell cycle and metastatic potential of tumor cells. Moreover, bioinformatics analysis has predicted its expression level as a potential prognostic biomarker of the response to immune therapy and potential therapeutic target (Xu et al., 2023). On the other hand, NEAT1 has been found upregulated in a cohort of patients with complete response to immune checkpoint inhibitors treatment, when compared with patients with no or partial response. NEAT1 upregulation has been associated with interferon-gamma signaling, cytokine production, and antigen presentation (Toker et al., 2023).

Many nuclear lncRNAs interact with chromatin regulators. Approximately 30% of lncRNAs have been associated with distinct chromatin regulatory complexes which include chromatin readers (chromobox proteins and transcription factors), writers (methyltransferases and acetyltransferases) and erasers (demethylases and deacetylases) (Guttman et al., 2011; Yang et al., 2022). lncRNAs recruit histone modifying complexes to chromatin and subsequently targeted loci are either activated or silenced, depending on the specific histone mark. Importantly, lncRNAs can provide regulatory specificity to chromatin regulatory complexes (Khalil et al., 2009; Guttman et al., 2011).

lncRNAs may regulate gene expression *in cis* or *in trans*. *cis*-acting lncRNA is defined as regulating the expression of neighboring gene on the same allele from which it has been transcribed, whereas *trans*-acting lncRNA is exported from transcription site, and may regulate distant genes within the same or other chromosome. Although some lncRNAs are *cis*-regulators, the vast majority of them function *in trans* (Ørom et al., 2010; Guttman and Rinn, 2012).

In conclusion, the advancement of bioinformatics analysis of genomic data and their public availability facilitates the prediction of novel non-coding RNAs along with their prognostic values in melanoma and other cancers (Li et al., 2023; Zhang et al., 2023). The contribution of lncRNAs to melanoma progression has been summarized elsewhere (Wozniak and Czyz, 2021; Montico et al., 2022; Dashti et al., 2023). Consequently, we will focus here only on those lncRNAs involved in gene silencing as binding partners of EZH2.

## 3 EZH2, a component of PRC2, acts as transcriptional repressor

**FIGURE 1:**
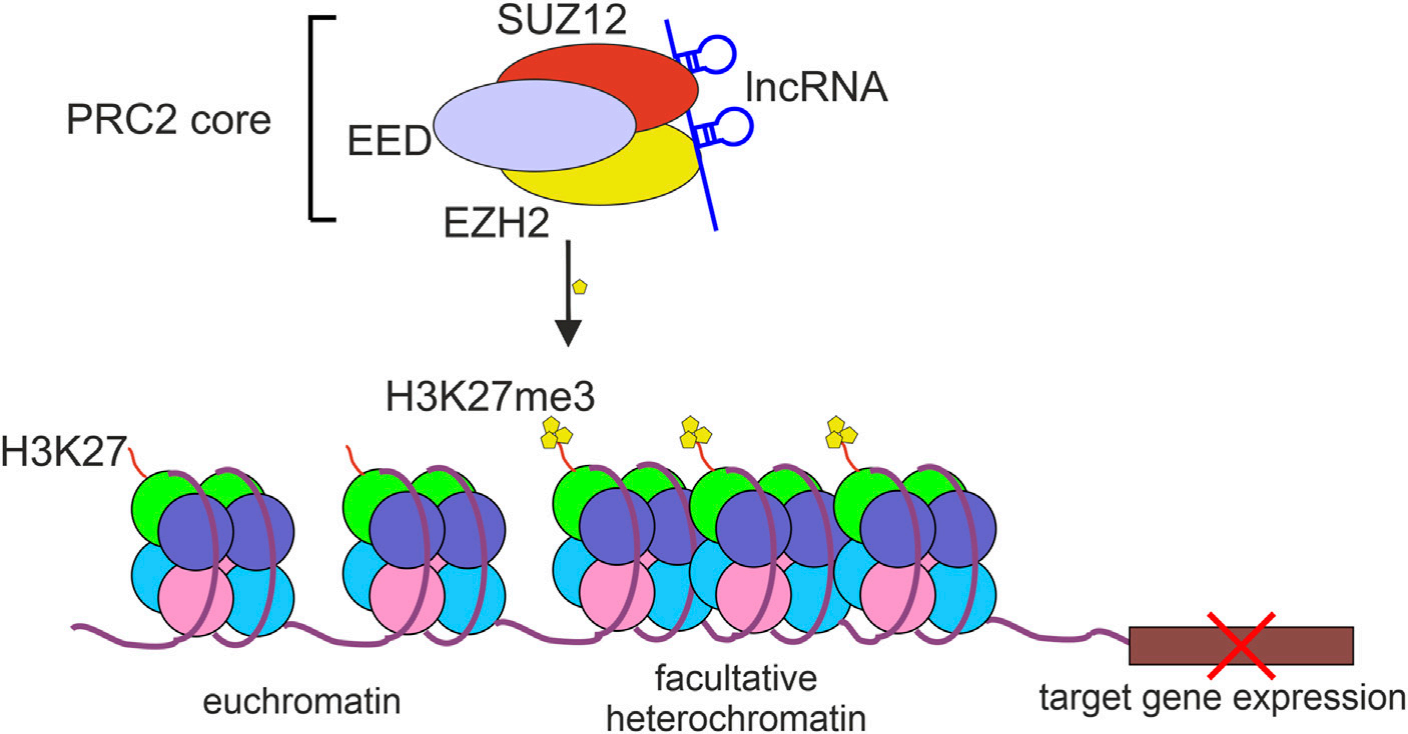
The repressive activity of PRC2 complex. The polycomb repressive complex 2 (PRC2) binds to and methylates histone 3 lysine 27 (H3K27me3) which results in chromatin compaction and repression of target gene expression. lncRNAs bind to PRC2 mostly through EZH2 or SUZ12 protein subunits.

EZH2 is a part of the polycomb repressor complex 2 (PRC2) that by modifying histones contributes to transcriptional repression of many genes (Sparmann and van Lohuizen, 2006). PRC2-mediated H3K27 trimethylation (H3K27me3) results in restricting the access to chromatin for RNA Polymerase II and various transcriptional regulators and transcription-associated factors (Liu, 2022). The core PRC2 complex contains at least three proteins: EZH2, embryonic ectoderm development (EED), and suppressor of zeste 12 homolog protein (SUZ12). Although EZH2 is considered to be a catalytic subunit of PRC2, the interaction of EZH2 with SUZ12 and EED is indispensable for PRC2 histone methyltransferase activity (Cao and Zhang, 2004) (Figure 1).

Emerging evidence indicates that EZH2 is contributing to progression of melanoma (Zingg et al., 2015; Mahmoud et al., 2016; Hoffmann et al., 2020). EZH2 and other PRC2 components are frequently amplified or overexpressed in melanoma, and several activating EZH2 mutations such as various mutations of tyrosine Y641 have been identified (Tiffen et al., 2016a; Hayward et al., 2017). This has correlated with DNA methylation and epigenetic silencing of melanoma tumor suppressors, including p16, p21, ciliary genes and AMD1 (Bachmann et al., 2006; Fan et al., 2011; Zingg et al., 2015; Zingg et al., 2018), but also genes involved in immune responses, including human leukocyte antigen (HLA) complex genes, several T cell-attracting chemokines, and interferon (IFN) response genes, interferon alpha-inducible protein 6 (IFI6) and interferon regulatory factor 6 (IRF9) (Tiffen et al., 2016a). High expression and activity of EZH2 have been linked to worse prognosis and shorter survival of melanoma patients (Bachmann et al., 2006). It has been reported that constitutive activity of ERK1/2 triggers increased EZH2 expression and global H3K27me3 levels in BRAF^V600^ melanoma cells (Yu et al., 2017; Grigore et al., 2020; Gebhardt et al., 2021). It has been shown that conditional knockout of EZH2, or inhibition of methyltransferase activity with small-molecule inhibitors of EZH2 lead to a remarkable reduction of proliferation of human melanoma cell lines and drastically diminish lymph node and lung metastases in mouse melanoma model (Zingg et al., 2015), and potentiate drug response in melanoma cell lines resistant to BRAF inhibitors (Uebel et al., 2023). Finally, recent research paper has depicted EZH2 as a key regulator of pigmentation and melanoma plasticity, since EZH2 expression negatively correlates with melanocyte inducing transcription factor (MITF) (Kuser-Abali et al., 2023). Pleiotropic and diverse roles of EZH2 prompted scientists worldwide to explore this methyltransferase as a promising target in the treatment of melanoma patients.

## 4 EZH2 as a key player in promoting melanoma resistance to immune and targeted therapies

Numerous reports indicate the involvement of EZH2 in regulation of tumor microenvironment (TME) and antitumor immune response, which directly affect immunotherapy efficacy (Fratta et al., 2013; Tiffen et al., 2016b; Kim et al., 2020; Sun et al., 2022). It has been shown in a mouse model of melanoma that treatment with anti-CTLA-4 antibody or interleukin 2 (IL-2) immunotherapy leads to persistent production of intratumoral tumor necrosis factor alpha (TNF-α) and T cell accumulation, which increases EZH2 expression (Zingg et al., 2017). Elevated EZH2 level has been associated with reduced expression of MHC class I genes and cytokine genes encoding CXCL9/10, which normally act to attract tumor-infiltrating lymphocytes (TILs) (Zingg et al., 2017). The authors of this report speculate that increased expression of EZH2 during melanoma immunotherapy with anti-CTLA-4 antibodies or IL-2 triggers diminution of therapy efficacy. Of note, in a mouse model of lung and colon carcinomas immunotherapy has failed to induce EZH2 expression, suggesting a melanoma-specific role of PRC2 in controlling immune evasion (Zingg et al., 2017). Increased expression of EZH2 has been associated with elevated level of Yin Yang 1 (YY1) transcription factor in melanoma TILs (Balkhi et al., 2018; Emran et al., 2019). It has been demonstrated that YY1 positively regulates the expression of checkpoint receptor proteins: PD-1, lymphocyte activation gene 3 (LAG-3) and T cell immunoglobin and mucin-domain containing 3 (TIM-3) and cooperates with EZH2 to silence the expression of IL-2 and IFN-γ in T cells (Balkhi et al., 2018). This leads to T cell exhaustion, a term describing defective CD8^+^ cell population that is hyporesponsive with reduced production of cytokines such as IFN-γ and TNF-α and various cytotoxic effectors (Apetoh et al., 2015). The T cell exhaustion state is also associated with hypomethylation of PD-1, LAG-3, and TIM-3 promoters (Emran et al., 2019). Downregulation of EZH2 in TILs via EZH2 small-molecule inhibitor CPI-1205 has been shown to augment their cytotoxic activity (Goswami et al., 2018). EZH2 is also known to play an important role in regulating the differentiation of CD4^+^ T cells and regulatory T cells (Tregs) (Tumes et al., 2013), which suppresses the immune response. It has been demonstrated in human tissues and murine models of different cancers, that EZH2 can increase and maintain Tregs stability by suppressing FOXP3 function (DuPage et al., 2015). Finally, EZH2 has been identified to inhibit NK cell activity by downregulating NKG2D receptors (Yin et al., 2015) and inhibit cytokine production from T helper type 1 (Th1) CD4^+^ T cells (Peng et al., 2015).

As mentioned previously, recently published research acknowledge EZH2 contribution to resistance to targeted therapy against BRAF^V600^ and MEK1/2 in melanoma. Gebhardt and others have noticed that miR-129-5p is among the most upregulated miRNAs during acute treatment with BRAF^V600^ inhibitors vemurafenib and dabrafenib, however, in melanoma cell lines with acquired resistance to BRAF^V600^ inhibitors (BRAFi) its expression diminishes (Gebhardt et al., 2021). miR-129-5p is a tumor suppressor that targets proliferation-inducing transcription factor SOX4 (Dai et al., 2017). The authors have speculated that EZH2 is responsible for the regulation of miR-129-5p/SOX4 axis, since EZH2-specific methyltransferase inhibitor tazemetostat triggered miR-129-5p diminution and reactivation of SOX4 in drug-resistant melanoma cell lines (Gebhardt et al., 2021). The same research group has further expanded their observations regarding EZH2 involvement in resistance to BRAFi and has found that combined treatment of BRAFi-resistant melanoma cell lines with tazemetostat and vemurafenib decreases cell viability, induces cell-cycle arrest and increases apoptosis, and this effect has been linked to the downregulation of polo-kinase 1 (PLK1), a key regulator of cell cycle and proliferation (Uebel et al., 2023). Increased levels of EZH2 and H3K27me3, observed specifically in melanoma cell lines resistant to vemurafenib or its analog PLX4720, have triggered silencing of melanoma tumor suppressor phosphatase and tensin homolog (PTEN) (Vidarsdottir et al., 2021). Finally, increased H3K27me3 level has been also identified in melanomas with mutated NRAS, second most frequent driver mutation in melanoma, and increased PRC2 activity has indirectly triggered the expression of mesenchymal markers and transcription factors such as zinc finger E-box binding homeobox 1 (ZEB1) and downregulation of epithelial protein E-cadherin (Terranova et al., 2021). Therefore, combined treatment with EZH2 and MEK1/2 inhibitors may provide a novel promising therapeutic strategy for NRAS-mutated and wild-type melanomas, as these types of melanomas are resistant to BRAF inhibitors (Terranova et al., 2021).

## 5 PRC2 and lncRNAs interactions

Various lncRNAs are able to bind several components of PRC2, which modify PRC2 target specificity. lncRNAs bound to the PRC2 complex components act as specific cellular ‘address codes’ that guide PRC2 silencing machinery to specific regions of the genome (Khalil et al., 2009; Batista and Chang, 2013; Nishikawa et al., 2017). Although the exact mechanism of lncRNA-EZH2 interaction with chromatin is elusive, some research suggest that lncRNAs preferentially bind to GA-rich DNA motifs in gene promoters to form RNA-DNA triplex, which functions as a platform to recruit PRC2 proteins (Mondal et al., 2015; Kalwa et al., 2016). Among three PRC2 core proteins EZH2 shows the highest affinity to lncRNAs, and lncRNA binding is feasible after EZH2 phosphorylation at threonine-350 (T350) mediated by cyclin-dependent kinases CDK1 and CDK2 (Chen et al., 2010; Zeng et al., 2011). Phosphorylation at T350 does not influence the assembly of the PRC2 complex, nor the histone methyltransferase activity, however it is important for the recruitment of PRC2 complex into its target genes (Chen et al., 2010). Moreover, studies in mice have revealed that T345 (corresponding to human T350) lies within EZH2 domain involved in the interaction with HOTAIR and Xist lncRNAs (Kaneko et al., 2010). In contrast, it has been demonstrated that phosphorylation of mouse EZH2 at T487 (human T492), also mediated by CDK1 and CDK2, promotes dissociation of EZH2 from active PRC2 complex, since this threonine residue is located in a domain responsible for SUZ12 binding. It has been reported that EZH2 phosphorylation at T487 in mouse suppresses histone methylation activity in differentiated mesenchymal stem cells and decreases cancer cell invasion (Wei et al., 2011).

Combination of high throughput sequencing with RNA immunoprecipitation (RIP-seq) has led to identification of more than 1,000 lncRNAs capable to bind EZH2 (Ye et al., 2020), and these associations were highly tissue- and context-dependent. Although many lncRNAs possess an EZH2-binding motif, this sequence has not been identified in all immunoprecipitated lncRNAs (Wang et al., 2018). First research on PRC2 binding to lncRNAs has identified Xist, a lncRNA that is required for X chromosome inactivation process (Brown et al., 1991). Xist binds directly to EZH2 and SUZ12 through the binding domain at the 5′ end of Xist transcript with a stretch of 8.5 repeats of a 28 nucleotide sequence forming a double hairpin called A-repeat domain (RepA), and allows the spreading of H3K27me3 mark *in cis* within X chromosome (Zhao et al., 2008). HOTAIR is another widely-recognized lncRNA binding partner of PRC2 complex. Tsai et al. (2010) identified the 300 bp secondary domain at the 5’ end of HOTAIR that binds to both EZH2 and SUZ12, Wu et al. (2013) have narrowed this sequence to a minimal, highly structured 89 nucleotide long domain required to bind the EZH2-EED heterodimer, and this binding is irrespective of EZH2 T350 phosphorylation. Unlike Xist, HOTAIR acts *in trans* and represses thousands of target genes throughout the whole genome (Rinn et al., 2007; Gupta et al., 2010). Both PRC2 binding sequences of Xist and HOTAIR differ significantly, that suggests the existence of several PRC2-lncRNA binding models corresponding to distinct functions of the PRC2 complex. The majority of lncRNAs that bind to EZH2 or SUZ12 do not show obvious or conservative binding motifs (Wang et al., 2018). Moreover, electrophoretic mobility shift assay (EMSA) and cross-linking experiments performed with mutated RepA sequences by Betancur and Tomari (2015) have suggested that the length of RNA molecule, and not the conservation of specific secondary structure of Xist RepA domain, is more important in binding to both EZH2 and SUZ12. Although both components of PRC2 complex bind lncRNA rather promiscuously, EED and JARID2 can increase the specificity of lncRNA-PRC2 binding (Cifuentes-Rojas et al., 2014; Kaneko et al., 2014). The exact factors that determine lncRNA-PRC2 binding specificity are still challenging to identify, due to the cryptic nature of these RNA-protein interactions, and failure to bind short or fragmented RNA molecules by PRC2 components (Betancur and Tomari, 2015).

## 6 EZH2–lncRNA interactions in melanoma

Several lncRNA-EZH2 interactions have been identified in melanoma and their involvement in melanoma invasion, metastasis, apoptosis or drug resistance have been confirmed by *in vitro* and/or *in vivo* experiments.

*CDKN2B*, encoding cyclin-dependent kinase inhibitor 2B (p15), is a tumor-suppressor gene located on chromosome 9p21. Competitive binding of p15 with CDK4 and CDK6 inhibits cell cycle progression from G1 phase to S phase (Lee and Yang, 2001). The expression of p15 has been found decreased in melanoma when compared with dysplastic nevi (Taylor et al., 2016). It has been reported that forkhead box 2 antisense 1 (FOXC2-AS1) lncRNA level increases with melanoma tumor stage, and its expression is negatively correlated with overall survival of melanoma patients (Xu et al., 2020a). It has been shown that although the majority of FOXC2-AS1 reside in cytoplasm, its nuclear interaction with EZH2 triggers downregulation of p15 *in trans*. FOXC2-AS1 silencing partially rescues p15 expression and induces apoptosis of melanoma cells (Xu et al., 2020a).

Expression level of long intergenic non-protein coding RNA, p53 induced transcript (LINC-PINT) has been detected significantly lower in melanoma tissues compared with adjacent normal tissues, and its low expression has been correlated with poor overall survival and disease free survival in melanoma patients (Xu et al., 2019). LINC-PINT predominantly resides in nucleus and binds EZH2, and its antitumor activity is related to PRC2-mediated epigenetic silencing of oncogenes such as CDK1, cyclin A2 (CCNA2), aurora kinase A (AURKA), and proliferating cell nuclear antigen (PCNA) (Xu et al., 2019). It has been found that overexpression of LINC-PINT in melanoma cell lines inhibits colony formation and cell migratory abilities, induces cell cycle arrest at G_0_/G_1_ *in vitro*, reduces tumor weight and inhibits lung metastases in nude mice model (Xu et al., 2019). LINC-PINT has been reported to bind EZH2 and SUZ12 through highly conserved among mammals, a consecutive guanine repeat sequence (Marín-Béjar et al., 2017).

The chromosome 6p22.3 cancer susceptibility candidate 15 (CASC15) locus is frequently amplified in metastatic melanoma tumors and cell lines, and upregulation of CASC15 expression has been associated with metastatic progression to brain metastases in a mouse xenograft model (Lessard et al., 2015). As CASC15 level has been found increased during melanoma progression, it might be considered as independent predictor of disease recurrence in patients with stage III lymph node metastasis (Lessard et al., 2015). It has been demonstrated that knockdown of CASC15 in melanoma cells inhibits Wnt/β-catenin signaling pathway, which results in decreased proliferation, invasion, and migration, as well as increased cell apoptosis (Sheng and Wei, 2020). Mechanistically, CASC15 binds to EZH2 and decreases the expression of tumor suppressor programmed cell death 4 (PDCD4) via increased H3K27me3 at PDCD4 promoter regions (Yin et al., 2018). CASC15 depletion represses proliferation, induces apoptosis and decreases invasion in melanoma cells and tumor growth *in vivo*, and this effect has been impaired after PDCD4 knockdown (Yin et al., 2018).

Focally amplified lncRNA on chromosome 1 (FALEC) has been identified as an oncogenic lncRNA in a variety of cancers, including melanoma. This lncRNA cooperates with EZH2 to negatively regulate the expression of p21 tumor suppressor (Ni et al., 2017), a product of *CDKN1A*. FALEC downregulation has been shown to trigger apoptosis, block EMT-like process and inhibit proliferation in melanoma cell lines (Ni et al., 2017). Moreover, FALEC expression may be used as an independent prognostic marker for melanoma patients, since its expression has been linked to metastasis, TNM (tumor, node, metastasis) stages and overall patients survival (Ni et al., 2017).

High expression in hepatocellular carcinoma (HEIH) lncRNA is an oncogenic lncRNA first identified in hepatocellular carcinoma cells by Yang et al. (2011). Its oncogenic properties have also been confirmed in melanoma cell lines and tissues (Zhao et al., 2017). It has been reported that HEIH knockdown inhibits proliferation, migration and invasion of melanoma cell lines (Zhao et al., 2017). Elevated expression of HEIH has correlated with advanced clinical stages of the disease and may serve as a predictor of poor outcome in melanoma patients (Zhao et al., 2017). The HEIH-EZH2 complex binds to the promoter of *miR-200* family and downregulates the expression of miR-200b, miR-200a and miR-429 (Zhao et al., 2017). In melanoma, the expression of miR-200 family members is involved in the regulation of cell proliferation, migration, invasion, EMT-like process and drug resistance (Liu et al., 2012; van Kempen et al., 2012). This miRNA cluster is also negatively regulated by lncRNA-EZH2 complex in other cancers (Sui et al., 2016).

Plasmacytoma variant translocation 1 (PVT1) is another lncRNA that represses the expression of *miR-200* family through EZH2-mediated binding to *miR-200c/141* family promoter (Chen et al., 2018). It has been shown that PVT1 expression is increased in melanoma tissues compared with benign nevi, and its expression can be a potential diagnostic biomarker and therapeutic target for melanoma (Chen et al., 2017a). PVT1 silencing inhibits proliferation, migration and invasion in melanoma cells, which is manifested by decreased expression of cyclin D, N-cadherin and vimentin oncogenes, and increased expression of E-cadherin (Chen et al., 2018).

Interleukin enhancer-binding factor 3-antisense RNA 1 (ILF3-AS1) has been found upregulated in melanoma tissues and cell lines, compared with normal counterparts, and its increased expression in melanoma patients is associated with metastatic characteristics and poor prognosis (Chen et al., 2017b). It has been demonstrated that the expression of ILF3-AS1 negatively correlates with *miR-200b/a/429* gene cluster. Mechanistically, ILF3-AS1 cooperates with EZH2 to *miR-200b/a/429* gene cluster promoter to block the expression of miR-200b, miR-200a and miR-429 miRNAs (Chen et al., 2017b).

Long intergenic non-coding RNA 01296 (LINC01296), also known as lymph node metastasis associated transcript 1 (LNMAT1), has been identified as a metastasis-promoting lncRNA in multiple cancer types. Its expression is higher in cutaneous and acral melanomas compared with benign nevi, and in melanoma with lymph node metastases vs*.* non-metastatic melanomas (Mou et al., 2019). It has been demonstrated that LNMAT1 promotes migration and invasion of melanoma *in vitro* and *in vivo* by cooperating with EZH2 to inhibit the expression of cell adhesion molecule 1 (CADM1), a recognized tumor suppressor that inhibits the expression of MMP-2 and MMP-9 matrix metalloproteinases (You et al., 2014). LNMAT1 depletion decreased EZH2 binding and H3K27me3 of the CADM1 promoter which results in CADM1 upregulation, and silencing CADM1 expression partially rescues inhibitory effects on melanoma cell migration and invasion induced by LNMAT1 depletion (Mou et al., 2019).

LncRNA miRNA-31 host gene (MIR31HG), formerly known as LOC554202, is 2,166 nucleotides long (Augoff et al., 2012). The expression of MIR31HG is significantly upregulated in melanoma tissues and cell lines, compared with their normal counterparts, and this upregulation is positively correlated with lymph node metastasis, distal metastasis, and TNM stage (Xu and Tian, 2020). It has been shown that in melanoma patients with BRAF^V600^ mutations the expression of MIR31HG negatively correlates with the expression of *CDKN2A*, encoding p16^INK4A^. Downregulation of p16^INK4A^ is mediated by MIR31HG, which directly interacts with SUZ12 and EZH2 to bind *CDKN2A* promoter (Montes et al., 2015). Knockdown of MIR31HG induces melanoma cell senescence, which is associated with re-expression of p16^INK4A^.

**FIGURE 2:**
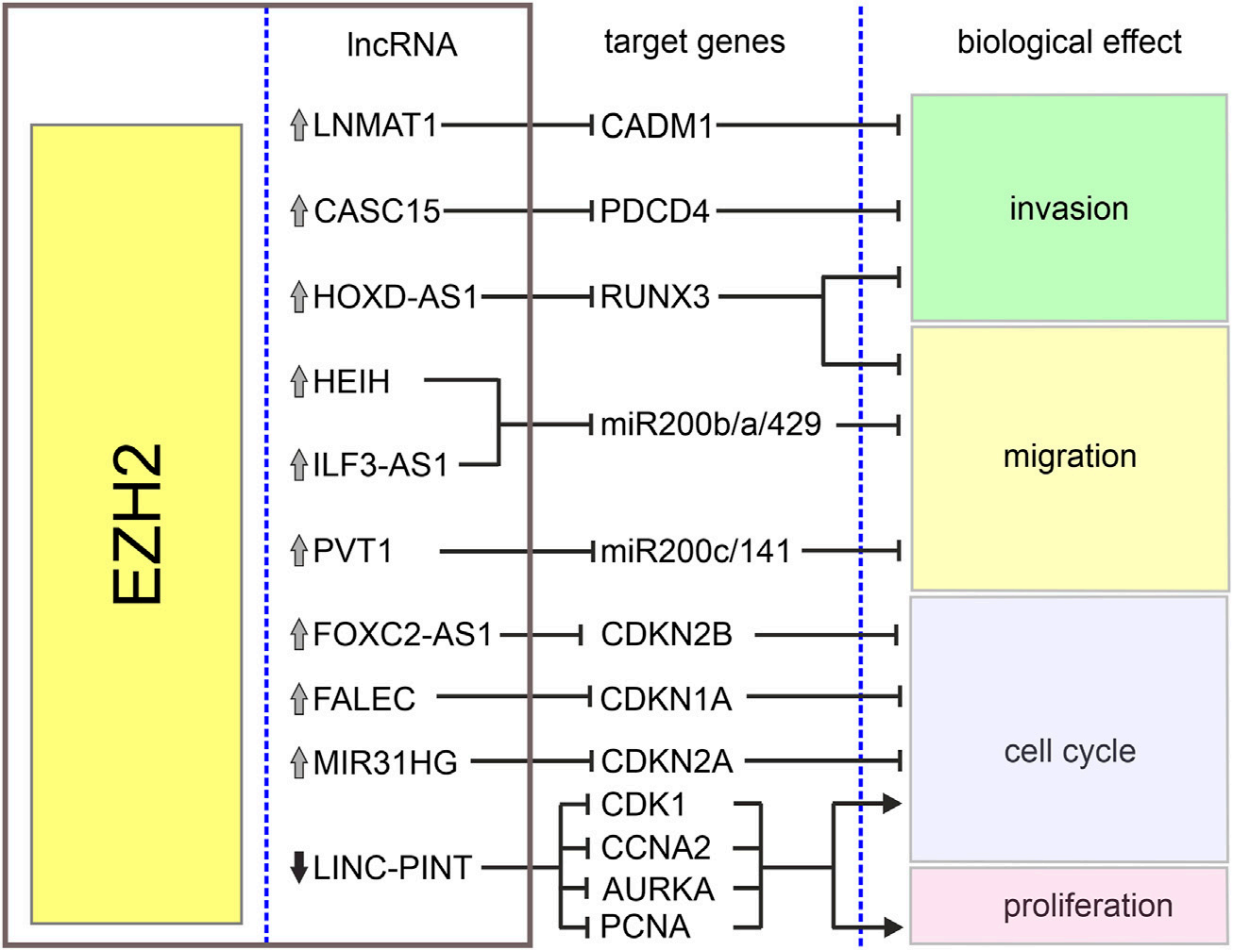
Biological effects of EZH2-lncRNA interactions in melanoma. The graph depicts interactions between EZH2, lncRNAs and their target genes in melanoma. Grey arrows denote upregulation and black arrow–downregulation of corresponding lncRNA. Sharp arrows and blunt arrows denote activation and inhibition of corresponding biological effect, respectively.

The homeobox cluster D antisense 1 (HOXD-AS1) is a lncRNA encoded in a cluster of *HOXD* genes and transcribed as an anti-sense transcript (Yarmishyn et al., 2014). This lncRNA has been identified as an oncogene in various tumors including gastric and liver cancers (Lu et al., 2017; Zheng et al., 2017). Its increased expression in melanoma when compared with normal tissues and cell lines has been associated with increased cell proliferation and invasion and poor overall survival of melanoma patients (Zhang et al., 2017). It has been reported that interaction between HOXD-AS1 and EZH2 contributes to downregulation of runt-related transcription factor 3 (RUNX3), a well-known suppressor of solid tumors (Kitago et al., 2009; Manandhar and Lee, 2018). It has been shown that HOXD-AS1 expression negatively correlates with the expression of RUNX3, and downregulation of RUNX3 attenuates inhibitory effects of HOXD-AS1 knockdown on proliferation and invasion (Zhang et al., 2017). Table 1 and Figure 2 summarize the cooperation between various lncRNAs and EZH2 in melanoma.

**TABLE 1:** EZH2-interacting lncRNA, their genomic location and target genes in melanoma. All listed lncRNA-EZH2 complexes act in melanoma nucleus *in trans*.

lncRNA	Genomic location	Expression in melanoma	Target gene(s)	Cell lines used	References
FOXC2-AS1	16q24.1	upregulated	*CDKN2B*	A375, sk-mel-110, MEL-RM, HEMn	Xu et al. (2020a)
LINC-PINT	7q32.3	downregulated	*CDK1, CCNA2, AURKA, PCNA*	A375, Mum2B, CRMM1, PIG1	Marín-Béjar et al. (2017), Xu et al. (2019)
CASC15	6p22.3	upregulated	*PDCD4*	A375, SK-MEL-2, M21, MEL-RM, B16, SK-MEL-1, HEMa-LP	Yin et al. (2018)
FALEC	1q21.2	upregulated	*CDKN1A*	M21, B16, MEL-RM, HEMn	Ni et al. (2017)
HEIH	5q35.3	upregulated	*miR200b/a/429*	SK-MEL-28, A375, A2058, SK-MEL-2, HEMa-LP	Zhao et al. (2017)
ILF3-AS1	19p13.2	upregulated	*miR200b/a/429*	SK-MEL-2, SK-MEL-28, A375, HEMa-LP	Chen et al. (2017b)
PVT1	8q24.21	upregulated	*miR200c/141*	A375, sk-mel-5, PIG1	Chen et al. (2018)
MIR31HG	9p21.3	upregulated	*CDKN2A*	BJ and TIG3 fibroblasts, TCGA SKCM samples	Montes et al. (2015)
LNMAT1	14q11.2	upregulated	*CADM1*	WM35, A375, A2058, B16F10, HEMa-LP	Mou et al. (2019)
HOXD-AS1	2q31.1	upregulated	*RUNX3*	B16, A375, A2508, HEMn	Zhang et al. (2017)

EZH2 expression can be also regulated by lncRNAs. For instance, growth arrest specific 5 (GAS5), a long non-coding lncRNA downregulated in advanced melanoma, recruits E2F transcription factor 4 (E2F4) to EZH2 promoter, which results in EZH2 repression in melanoma, and this repression correlates with the upregulation of cyclin-dependent kinase inhibitor 1C (CDKN1C) (Xu et al., 2020b). GAS5 has been previously identified as tumor suppressor in melanoma. It has been demonstrated that the expression of GAS5 positively correlates with the expression of miR-137 (Bian et al., 2017). This miRNA has been found to suppress the expression of cMYC, Y-box binding protein 1 (YB1), MITF and EZH2 in melanoma (Luo et al., 2013).

## 7 Targeting EZH2 and lncRNA-EZH2 interactions in cancer

Since EZH2 and numerous lncRNAs have been already linked to tumorigenesis, EZH2 is becoming an emerging target for the treatment of various solid cancers. While in this review we focus on lncRNAs-EZH2 interaction, mainly in melanoma, diverse approaches to modulate the levels of specific lncRNAs or attempts to decrease EZH2 activity in melanoma have been described in other review papers (Tiffen et al., 2015a; Wozniak and Czyz, 2021). Several specific EZH2 small molecule inhibitors that target its methyltransferase activity, such as tazemetostat, valemetostat, GSK503 or GSK126 have been discovered or synthesized (Zhang et al., 2022). Tazemetostat (EPZ-6438) has been the first EZH2 inhibitor clinically approved by the Food and Drug Agency (FDA) to treat metastatic epithelioid sarcoma (Hoy, 2020). Since then, many phase I/II clinical trials have been evaluated for EZH2 inhibition in other tumors (Eich et al., 2020). According to clinicaltrials.gov site (as for 12 May 2023), there are in total 60 clinical trials regarding EZH2 inhibition in cancer (active, recruiting or completed), but only two in melanoma: tazemetostat as an adjuvant to the targeted therapy with dabrafenib and trametinib (NCT04557956), and lirametostat (CPI-1205), another EZH2 inhibitor, combined with CTLA-4 inhibitor ipilimumab (NCT03525795). So far, no inhibitors or clinical trials regarding the blockade of specific lncRNA-EZH2 interactions have been designed.

However, since EZH2 orchestrates global gene expression, undesirable off-targets and side-effects of EZH2 inhibition might become a clinical problem. EZH2 directly interacts with histone deacetylases (Simon and Lange, 2008; Zhang et al., 2015) and DNA methylases (Viré et al., 2006; Liu et al., 2018; Tiffen et al., 2020), and its non-canonical activity comprises methylation and thus activation of non-histone proteins, such as signal transducer and activator of transcription 3 (STAT3) in glioblastoma (Kim et al., 2013) or androgen receptor (AR) in castration-resistant prostate cancer (Xu et al., 2012; Kim et al., 2018), and this activity is PRC2-independent. EZH2 directly interacts with other proteins such as breast cancer gene 1 (BRCA1) binding to EZH2 in breast cancer cells (Wang et al., 2013). EZH2 T350, a residue important for lncRNA binding, is localized in BRCA1-binding domain. BRCA1-EZH2 interaction blocks lncRNA binding to EZH2. It has been reported that decreased expression of BRCA1 triggers genome-wide EZH2 retargeting and elevates H3K27me3 levels which blocks embryonic cell differentiation and enhances breast cancer migration and invasion (Wang et al., 2013). Also, EZH1, a typical component of PRC1 complex, may compensate for PRC2 activity in case of EZH2 inhibition (Shen et al., 2008). EZH2 is expressed only in actively dividing cells whereas EZH1 is present mostly in differentiated, non-dividing populations (Bracken et al., 2003; Shen et al., 2008; Margueron and Reinberg, 2011). EZH1 is identical to EZH2 in 65% (Simon and Kingston, 2009) and both proteins exhibit similar histone methyltransferase activity (Shen et al., 2008). However, unlike EZH2 silencing, knockdown of EZH1 does not influence global H3K27me3 redistribution (Margueron et al., 2008). EZH1 only partially complements EZH2 activity in maintaining pluripotency during embryonic stem cells differentiation (Shen et al., 2008). Moreover, EZH1 lacks all threonine residues that are phosphorylated by CDKs in EZH2, therefore, the EZH1-mediated lncRNA recruitment to PRC2 is significantly impeded (Kaneko et al., 2010). Finally, EZH2 activity in cancer is modulated by various oncogenic mutations in *EZH2* (Tiffen et al., 2015b; Zingg et al., 2015; Souroullas et al., 2016) and post-translational modifications of components of the PRC2 complex (Li et al., 2020; Yang and Li, 2020), which might influence the output of EZH2-targeted therapies. However, these aforementioned issues go beyond the topic of this review.

An interesting approach to directly target lncRNA–EZH2 interactions in different cancers has been proposed by two research teams. A high-throughput screening for small molecules inhibiting the RNA-protein interaction may lead to identification of either: i) small-molecule inhibitors that directly block the EZH2 RNA-binding pocket, or ii) molecules that bind to RNA and change the spatial structure of lncRNA to prevent EZH2 binding. Second option would lead to identification of highly lncRNA-specific compounds that might act selectively to block only the specific EZH2-lncRNA binding, without affecting other EZH2 activities. With the use of the high-throughput method adapted from AlphaScreen technology, that has been used previously to monitor RNA-protein binding and for screening of small molecules targeting these interactions (Mills et al., 2007), Pedram Fatemi et al. (2015) have identified several small-molecule inhibitors of the interaction between EZH2 and two lncRNAs: HOTAIR and brain-derived neurotrophic factor antisense (BDNF-AS) in different cancers. With the use of 3D modeling prediction programs enabling identification of several hairpin loop structures in the HOTAIR molecule that might serve as docking sites for several small-molecule compounds, followed by *in silico* high-throughput screening to find interacting ligands, Ren et al. (2019) identified a compound AC1NOD4Q that selectively blocks the HOTAIR-EZH2 interaction in glioma and breast cancer cell lines. This strongly suggests that targeting individual dysregulated lncRNAs or interaction with their protein partners could be a cornerstone of cancer precision medicine in the nearest future.

## 8 Final conclusion

This review demonstrates that several lncRNAs contribute to melanoma progression via direct interaction with EZH2. Since EZH2 plays an important role in epigenetic regulation, its aberrant expression and activity modulated by lncRNAs are highly relevant to cancer and other diseases. Improving our understanding regarding the role of lncRNA-EZH2 interaction network in melanoma would help to develop novel therapeutic strategies.
